# Latent Fingermark Development Using CVD-Synthesized Two-Dimensional GaS_*x*_Te_1−*x*_ Alloy Nanosheets

**DOI:** 10.3390/molecules31162912

**Published:** 2026-08-20

**Authors:** Runkai Hu, Jun Zhu, Fang Zhou, Yue Zhou, Shangqi Feng, Ziyin Zhang, Yujing Zhao, Feiya Fu

**Affiliations:** 1 College of Investigation, People’s Public Security University of China, Beijing 100038, China; 2 Department of Criminal Science and Technology, and Department of Foundation Course, Hunan Police College, Changsha 410138, China; 3 School of Physics and Electronics, Synergetic Innovation Center for Quantum Effects and Application, Key Laboratory of Low-Dimensional Quantum Structures and Quantum Control of Ministry of Education, and Key Laboratory for Matter Microstructure and Function of Hunan Province and Institute of Interdisciplinary Studies, Hunan Normal University, Changsha 410081, China; 4 School of Electrnic Information and Physics, Central South University, Changsha 410083, China; 5 School of Physics, Electronictechnology and Intelligent Manufacturing, Huaihua University, Huaihua 418008, China; 6 Department of Chemistry and Biochemistry, California State University Los Angeles, 5151 State Univ Dr, Los Angeles, CA 90032, USA

**Keywords:** GaS_*x*_Te_1−*x*_ alloy nanosheets, chemical vapor deposition, photoluminescence, latent fingermark development, fluorescence contrast

## Abstract

Two-dimensional GaS_*x*_Te_1−*x*_ alloy nanosheets with different compositions were synthesized by chemical vapor deposition using GaS and GaTe powders as precursors. Scanning electron microscopy (SEM) and energy-dispersive X-ray spectroscopy (EDS) analyses confirmed their sheet-like morphology and the uniform distribution of S and Te, while Raman and photoluminescence measurements revealed composition-dependent vibrational and emission characteristics. Te-rich samples exhibited position-dependent emission ranging from the red to the near-infrared region, whereas increasing the S content gradually shifted the emission toward the blue-green region. Among the synthesized samples, GaS_0.9_Te_0.1_ showed a relatively stable photoluminescence peak near 520 nm and was therefore selected as a fluorescent powder for latent fingermark development. Its performance was evaluated on glass, stainless steel, plastic, and ceramic surfaces and compared with that of silver powder, gold powder, and commercial red fluorescent powder. GaS_0.9_Te_0.1_ produced clear fluorescent ridge patterns and strong background contrast, particularly on glass, plastic, and white ceramic. The mean contrast across the four substrates reached 25.83, exceeding that of the reference powders. These results demonstrate that GaS_*x*_Te_1−*x*_ nanosheets possess tunable optical properties and that GaS_0.9_Te_0.1_ is a promising fluorescent material for latent fingermark development on non-porous surfaces.

## 1. Introduction

In recent years, two-dimensional layered materials have attracted considerable attention due to their unique electronic structures, optical responses, and surface properties. Layered materials can be prepared as thin nanosheets with large lateral dimensions by mechanical exfoliation, liquid-phase exfoliation, or chemical vapor deposition, providing an important platform for investigating physical properties and device applications under low-dimensional conditions [1,2,3,4]. Graphene, as a representative honeycomb two-dimensional system, exhibits a typical Dirac-type electronic structure and excellent carrier transport properties [5]. However, pristine graphene lacks an intrinsic bandgap, which hinders effective current switching and wavelength-selective photoresponse, thereby limiting its direct application in semiconductor and optoelectronic devices. Consequently, two-dimensional layered semiconductors with intrinsic bandgaps, including transition metal dichalcogenides and group III monochalcogenides, have gradually become major research focuses [6,7,8,9,10,11].

Latent fingerprints are typically developed using conventional powders such as silver powder and gold powder, which rely primarily on color contrast against the substrate background. Their development performance is often compromised by substrate color, surface reflectance, and non-selective powder deposition. Although conventional phosphors can improve observation conditions, they may still suffer from background fluorescence, particle aggregation, or uneven ridge coverage. Therefore, novel powder materials suitable for latent fingerprint development should not only establish adequate contact with fingerprint residues but also exhibit strong and tunable optical signals to balance ridge adhesion and background suppression.

Two-dimensional layered semiconductors generally possess pronounced structural anisotropy: adjacent layers are connected mainly by weak van der Waals forces, while intralayer atoms are bonded through relatively strong covalent bonds [12,13,14,15,16,17,18]. This bonding configuration facilitates the formation of ultrathin lamellar structures and provides high specific surface areas. A large specific surface area implies that a unit mass of material can expose more surface sites and achieve more extensive contact with external substances; thus, such materials have been widely applied in photocatalysis, electrocatalysis, gas adsorption, chemical sensing, and other interfacial processes [19,20,21,22,23,24,25]. Related selenide and telluride nanostructures have exhibited diverse structural and functional characteristics with respect to morphology control [26], Raman vibrations [27,28], topological transport [29], thermoelectric performance [30], and biomedical applications [28,30]. Among group III monochalcogenides, GaS features a typical layered structure and favorable photoresponse properties; few-layer and ultrathin GaS have been utilized to construct highly sensitive photodetectors with notable photoelectric responses on both rigid and flexible substrates [26,31]. GaTe, as an important III–VI van der Waals semiconductor, has been primarily investigated for applications in photodetectors and radiation detection [32,33].

Tuning the band structure and luminescence properties is essential for expanding the application scope of semiconductor materials. In addition to thickness control and heterostructure construction, ligand exchange, defect modulation, interface modification, nanostructure design, and compositional alloying can effectively modulate the energy-level structure and optical response of materials [6,34,35,36]. Among these strategies, alloying enables continuous compositional adjustment by varying elemental ratios, thereby extending the ranges of bandgaps and luminescence wavelengths while preserving the host crystal structure.

For latent fingermark development, the contrast between ridges and the background is often low, as the ridges consist of trace amounts of sweat and sebum deposited during contact. Conventional fingermark development powders rely primarily on color contrast against the substrate background, but their effectiveness is affected by substrate color, surface reflectance, and non-selective powder deposition [37,38]. Moreover, most fingermark development powders exhibit granular morphologies, whereas two-dimensional sheet-like structures offer larger specific surface areas than granular counterparts. On this basis, GaS_*x*_Te_1−*x*_ alloy nanosheets were selected as latent fingermark development materials in this study [39,40]. GaS and GaTe share similar layered structures, facilitating S/Te ratio modulation while preserving the lamellar morphology; their differences in bandgap and luminescence properties enable composition-dependent optical responses. Compared with GaS or GaTe, which have fixed emission wavelengths, GaS_*x*_Te_1−*x*_ offers greater spectral tunability, allowing the selection of more suitable observation wavelengths for substrates with varying colors, reflectances, and background fluorescence.

**Figure 1:**
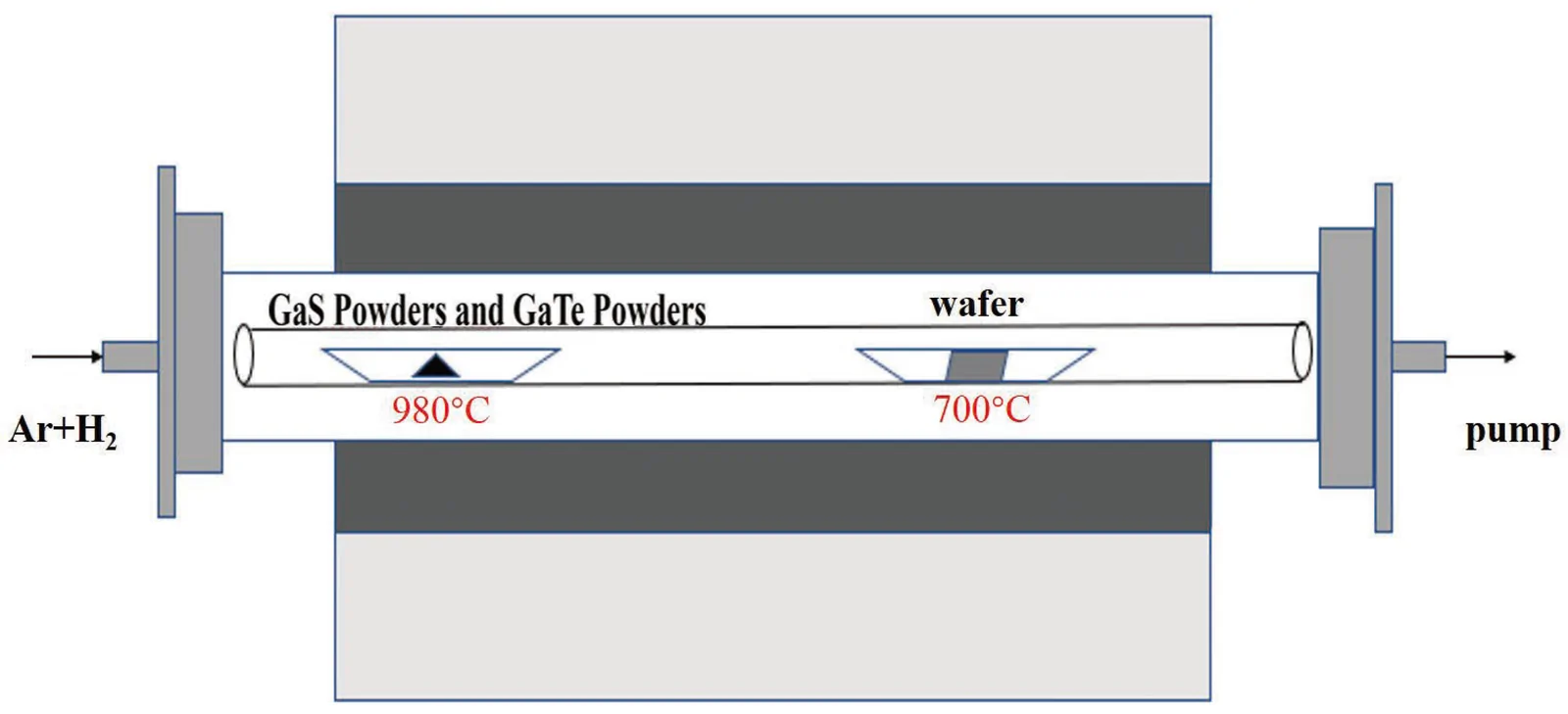
Schematic illustration of the CVD synthesis of GaS_*x*_Te_1−*x*_ alloy materials.

In this study, GaS_*x*_Te_1−*x*_ alloy nanosheets with different compositions were synthesized by chemical vapor deposition in Figure 1. Their morphology, elemental distribution, and composition-dependent optical properties were characterized by scanning electron microscopy, energy-dispersive X-ray spectroscopy, Raman spectroscopy, and photoluminescence spectroscopy. The resulting materials were then applied to the development of latent fingermarks on various non-porous substrates and qualitatively compared with silver powder, gold powder, and a commercial fluorescent powder. Their performance was evaluated in terms of ridge continuity, detail recognizability, background interference, and fluorescence contrast, providing experimental support for the application of composition-tunable 2D fluorescent materials in latent fingermark development [38,41,42].

## 2. Experimental Sections

### 2.1. Preparation of GaS_x_Te_*1*−x_ (0⩽x⩽1) Micro-Nanosheet

GaS_*x*_Te_1−*x*_ alloy nanosheets were synthesized via chemical vapor deposition (CVD). Commercial GaS and GaTe powders (purity 99.999%; Jiangxi Ketai Advanced Materials Co., Ltd., Gao’an, China) were used as precursors. The GaS:GaTe molar ratios were set to 0.1:0.9, 0.2:0.8, 0.3:0.7, 0.4:0.6, 0.5:0.5, 0.6:0.4, 0.7:0.3, 0.8:0.2, and 0.9:0.1. The total precursor mass was 4.0 g for each growth experiment. Single-side polished p-type SiO_2_/Si wafers (<100> orientation, SiO_2_ thickness 300 nm) were used as growth substrates. CVD growth was performed in a tube furnace (CVD(D)-04/60/3, Risine, Hefei, China) equipped with a quartz tube approximately 150 cm in length, with an inner diameter of 25 mm and an outer diameter of 30 mm. The thoroughly mixed GaS/GaTe precursor powders were placed in a ceramic boat positioned in the high-temperature source zone, while the cleaned SiO_2_/Si substrates were placed in another ceramic boat in the downstream deposition zone. The distance between the precursor boat and the substrate boat was approximately 20 cm. Prior to growth, the source zone and deposition zone of the furnace were preheated to 980 °C and 700 °C, respectively, and held at these temperatures for 2 h to stabilize the temperature distribution. Since the furnace had already stabilized at the target temperatures before the reaction components were introduced, no programmed temperature ramping was applied during the growth stage. Before growth, the quartz tube was purged with high-purity argon to remove residual air. During growth, high-purity Ar and H_2_ (99.9999%) were continuously introduced at flow rates of 100 SCCM and 25 SCCM, respectively. A mechanical pump operated continuously throughout the growth process, and the deposition time was 2 h. After growth, the furnace heating was turned off, and the system was allowed to cool naturally to room temperature without programmed cooling. The SiO_2_/Si substrates with deposited GaS_*x*_Te_1−*x*_ nanosheets were then removed from the ceramic boat in the deposition zone for subsequent characterization.

### 2.2. Sample Characterization

The size and morphology of the final products were examined using scanning electron microscopy (SEM, FEI NovaSEM-450, Hillsboro, OR, USA). Chemical composition and elemental distribution were analyzed by energy-dispersive X-ray (EDX) spectroscopy. The elemental contents were determined using inductively coupled plasma optical emission spectrometry/mass spectrometry (ICP-OES/MS; Agilent 7800, Santa Clara, CA, USA). The cross-sectional thickness of the samples was measured by atomic force microscopy (AFM; FM-Nanoview 800, Suzhou, China). Raman spectra were recorded using a high-resolution Raman spectrometer (LabRAM HR Evolution, Horiba, Longjumeau, France) with a continuous-wave laser operating at an excitation wavelength of 532 nm and a power of 5 mW. The photoluminescence quantum yield (PLQY) was measured using a steady-state and transient fluorescence spectrophotometer (FLS1000, Edinburgh Instruments, Livingston, UK).

### 2.3. Preparation and Deposition of Latent Fingermark Samples

To minimize inter-donor variability, all comparative fingermark development experiments were conducted using sebaceous latent fingermarks from the same 22-year-old donor. Prior to deposition, the donor washed their hands as usual and allowed them to air-dry. The fingertip was then gently pressed onto the substrate for approximately 3 s under similar deposition conditions. The deposition pressure was not quantitatively measured. To reduce variability introduced by fingermark deposition, all fingermarks were deposited by the same donor using a standardized light-pressure procedure, with approximately consistent contact pressure and duration. For each experimental condition, five independently deposited fingermarks were prepared as replicates. After deposition, the fingermarks were subjected to powder development within 60 min. Unless otherwise stated, the same fingermark preparation protocol was used for all qualitative and quantitative experiments.

All developed fingermarks were photographed using a digital camera (EOS 700D, Canon, Tokyo, Japan) under controlled imaging conditions. A multi-wavelength light source (CY-7032B, YIXIONG, Yueqing, China) was used as the illumination source for fingermark development, including white light and 355 nm wavelength light. No additional emission filters were used. The camera was operated in manual mode with an exposure time of 1/4 s, an aperture of f/8, and ISO 200. The distance from the camera to the sample was fixed at 15 cm, and the imaging geometry was kept constant during quantitative comparisons.

### 2.4. Qualitative and Quantitative Evaluation of Fingermark Development Performance

To comprehensively evaluate the latent fingermark development performance of GaS_0.9_Te_0.1_ alloy nanosheets, the analysis considered two aspects: the degree of fingermark feature recovery and local image contrast.

#### 2.4.1. Qualitative Analysis and Evaluation of Latent Fingerprint Development

Four fingermark development powders were selected, and sebum-rich and oil-rich latent fingermarks were deposited on four types of substrates for comparison in terms of overall ridge pattern integrity, ridge continuity, background interference, and the recovery of detailed features.

Referring to related studies [43], fingermark features were classified into three levels: level 1 features (overall pattern and ridge flow), level 2 features (minutiae such as endpoints and bifurcations), and level 3 features (microstructures including sweat pores and ridge edge morphology). Higher-level features are more difficult to visualize and identify; thus, the recovery of higher-level features better reflects the fine-detail development capability of the material. According to relevant forensic identification standards, the clear display of no fewer than eight level 2 minutiae is considered an important reference for determining whether a fingerprint has comparison value [44].

#### 2.4.2. Quantitative Analysis of Local Contrast

To quantitatively evaluate the development performance of different powders on rough non-porous substrates, the local contrast between ridges and furrows was used as the evaluation metric.

First, color fingermark images were imported into Adobe Photoshop and converted to grayscale. When the grayscale value of the ridges was lower than that of the furrows, the image was inverted to ensure that peaks in the subsequent grayscale profile corresponded to ridges and valleys corresponded to furrows. Subsequently, the image was imported into Image J 1.54r, and a straight line perpendicular to the local ridge flow was drawn using the Straight Line tool, crossing no fewer than 10 sets of ridges and furrows. The grayscale intensity profile was then extracted using the “Analyze–Plot Profile” function [45].

The grayscale profile was imported into Origin, and the peak and valley regions were identified based on the original image. The integrated area of each region was calculated, and the ratio of the integrated area to the corresponding interval length was used to obtain the average grayscale intensity [42]. The average grayscale intensities of the ridge and furrow regions were recorded as I_*r*_ and I_*v*_, respectively, and the local contrast was calculated according to Equation (1) [43]:

(1) $$\begin{array}{c} D=\left|I_{v}-I_{r}\right| \end{array}$$

To minimize differences caused by region selection, the ridge–furrow analysis regions were selected according to a uniform standard for all images. For each experimental condition, five independently deposited, developed, photographed, and quantitatively analyzed sebaceous latent fingermarks were used (n = 5). Each independently deposited fingermark was considered one experimental replicate. Local contrast values were calculated for each fingermark image, and the five fingermark-level contrast values were then used for statistical analysis. Results are presented as the mean ± standard deviation (SD). One-way analysis of variance (ANOVA) was used to assess differences among the four development powders on each substrate, followed by Tukey’s honestly significant difference (HSD) post hoc multiple comparison test. A *p*-value < 0.05 was considered statistically significant.

## 3. Results and Discussion

### 3.1. Characterization of GaS_x_Te_*1−*x_ (0⩽x⩽1) Alloy Micro-Nanosheets

**Figure 2:**
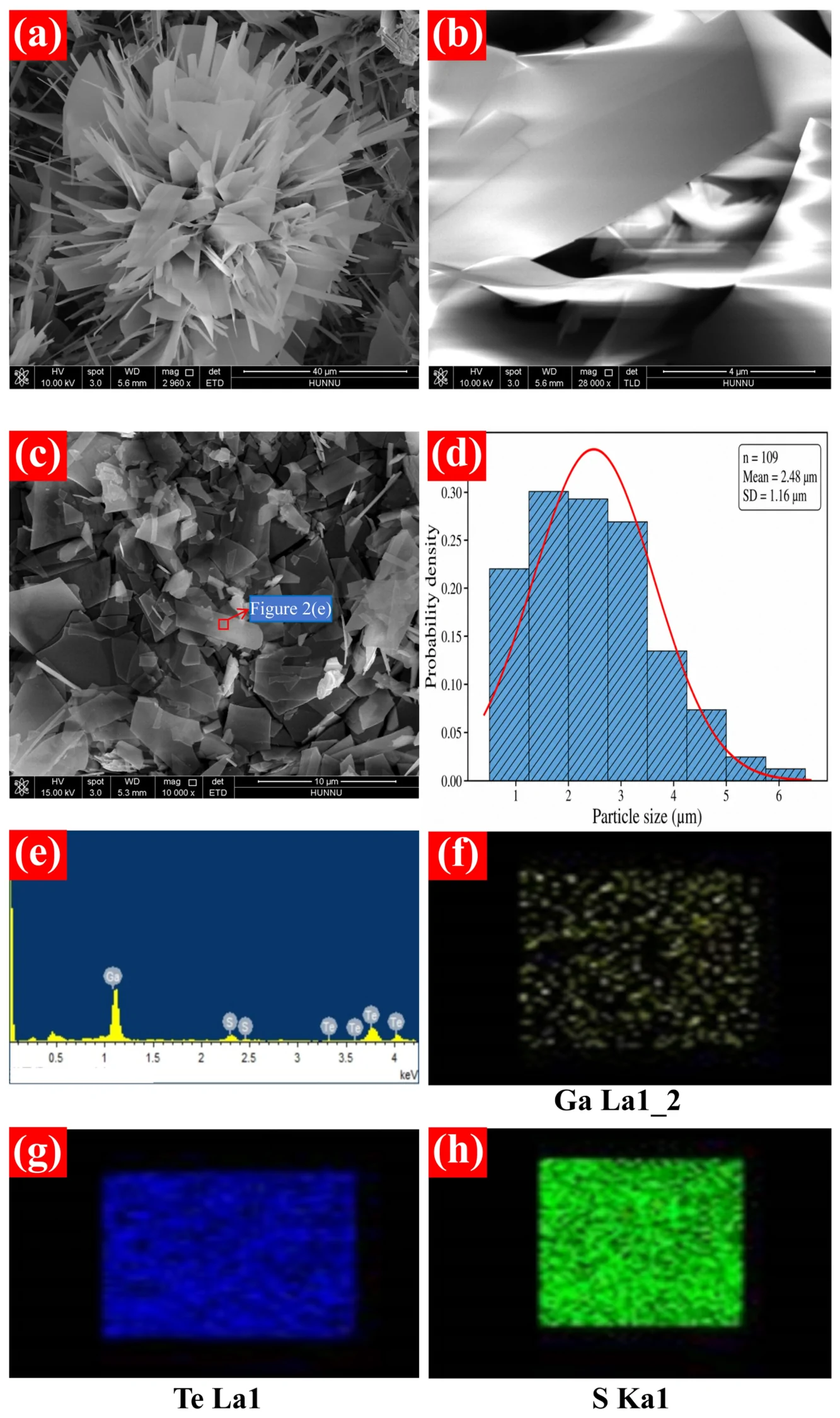
(**a**,**b**) SEM images of GaS_*x*_Te_1−*x*_ alloy nanosheets; (**c**) SEM image of GaS_*x*_Te_1−*x*_ alloy nanosheets after grinding; (**d**) particle size distribution of GaS_*x*_Te_1−*x*_ alloy nanosheets after grinding; (**e**–**h**) EDS spectrum and elemental mapping of Ga, Te, and S for a single GaS_*x*_Te_1−*x*_ alloy nanosheet.

The morphology and elemental distribution of the synthesized GaS_*x*_Te_1−*x*_ micro-/ nanosheets were characterized by SEM and EDS. As shown in Figure 2a, the obtained products exhibited a sheet-like morphology rather than a monolayer structure. Figure 2b displays a representative nanosheet with a lateral size exceeding 10 μm. After grinding, the GaS_0.9_Te_0.1_ material retained a predominantly sheet-like morphology but was broken into micron-scale particles, as shown in Figure 2c. Image analysis using the maximum Feret diameter yielded an average apparent lateral size of approximately 2.48 ± 1.16μm, as shown in Figure 2d. It is evident that the majority of particle sizes are significantly smaller than the characteristic dimensions of friction ridges and sweat pores. This size relationship facilitates localized deposition of the powder along fingerprint residues while minimizing geometric occlusion of fine features such as ridge edges and sweat pores. Figure 2e presents the EDS spectrum of GaS_0.2_Te_0.8_, with corresponding elemental mapping images shown in Figure 2f–h. The elemental mapping results reveal that Ga, S, and Te are spatially co-distributed within the nanosheets. As summarized in Table 1, combined with the bulk elemental compositions obtained by ICP-MS, these results collectively confirm that both S and Te have been incorporated into the synthesized Ga–S–Te nanosheets.

**Figure 3:**
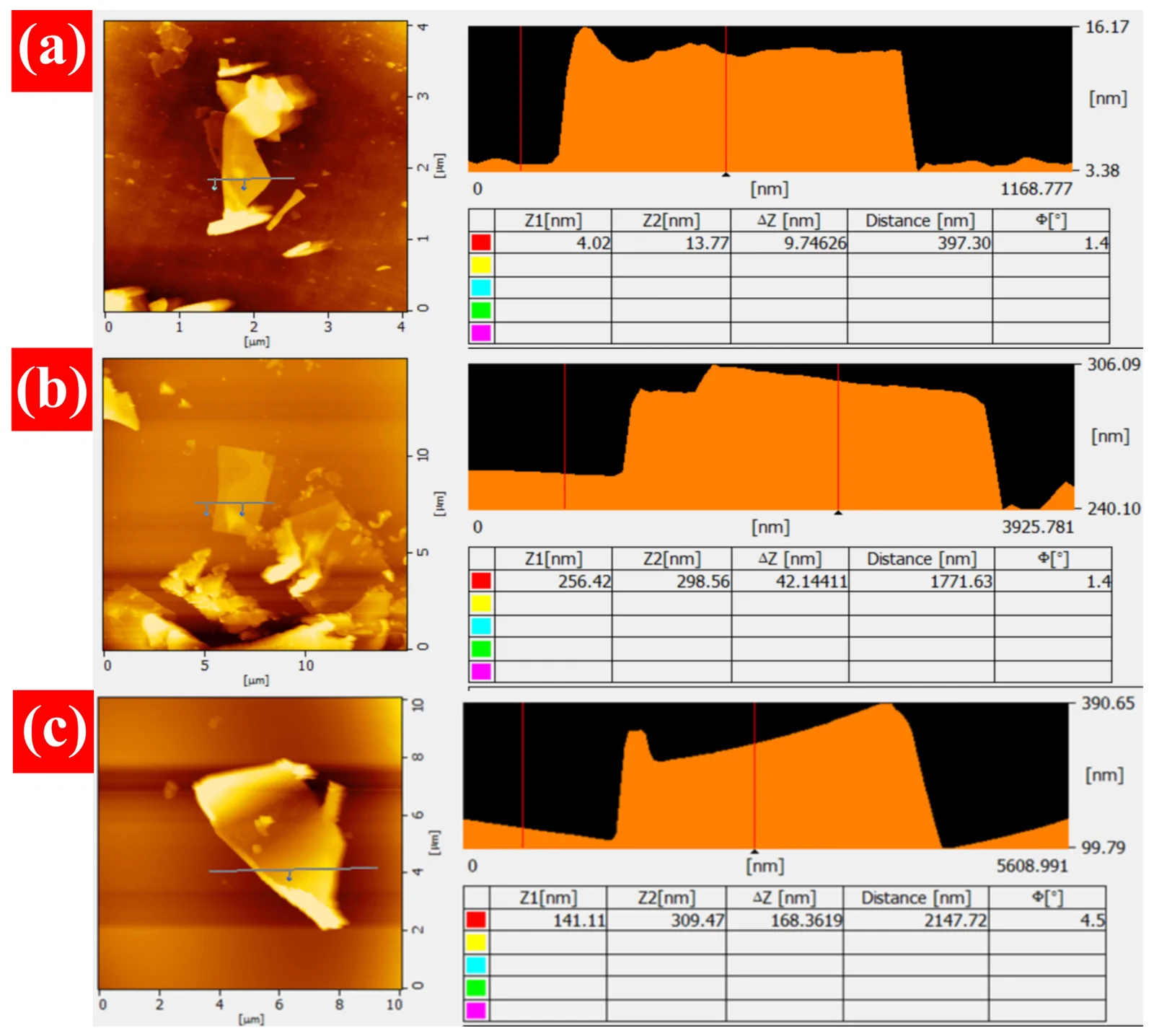
AFM topographic images of GaS_*x*_Te_1−*x*_ nanosheets collected from different positions in the deposition zone: (**a**) flakes collected from the front one-third of the deposition zone, with a thickness of approximately 10 nm; (**b**) flakes collected from the middle one-third of the deposition zone, with a thickness of approximately 40 nm; (**c**) flakes collected from the rear one-third of the deposition zone, with a thickness of approximately 170 nm.

To further determine the thickness of the GaS_*x*_Te_1−*x*_ nanosheets, AFM measurements were performed on representative flakes collected from three positions along the deposition zone of the ceramic boat (corresponding to the front third, middle third, and rear third), as shown in Figure 3. The height profiles revealed thicknesses of approximately 10, 40, and 170 nm, respectively. Combined with the micron-scale lateral dimensions observed by SEM, these results confirm that the obtained products exhibit a typical sheet-like morphology characterized by micron-scale lateral dimensions and nanometer-scale thicknesses.

### 3.2. Raman Characteristics of GaS_x_Te_*1−*x_ (0.1⩽x⩽0.9) Alloy Micro-Nanosheets

**Figure 4:**
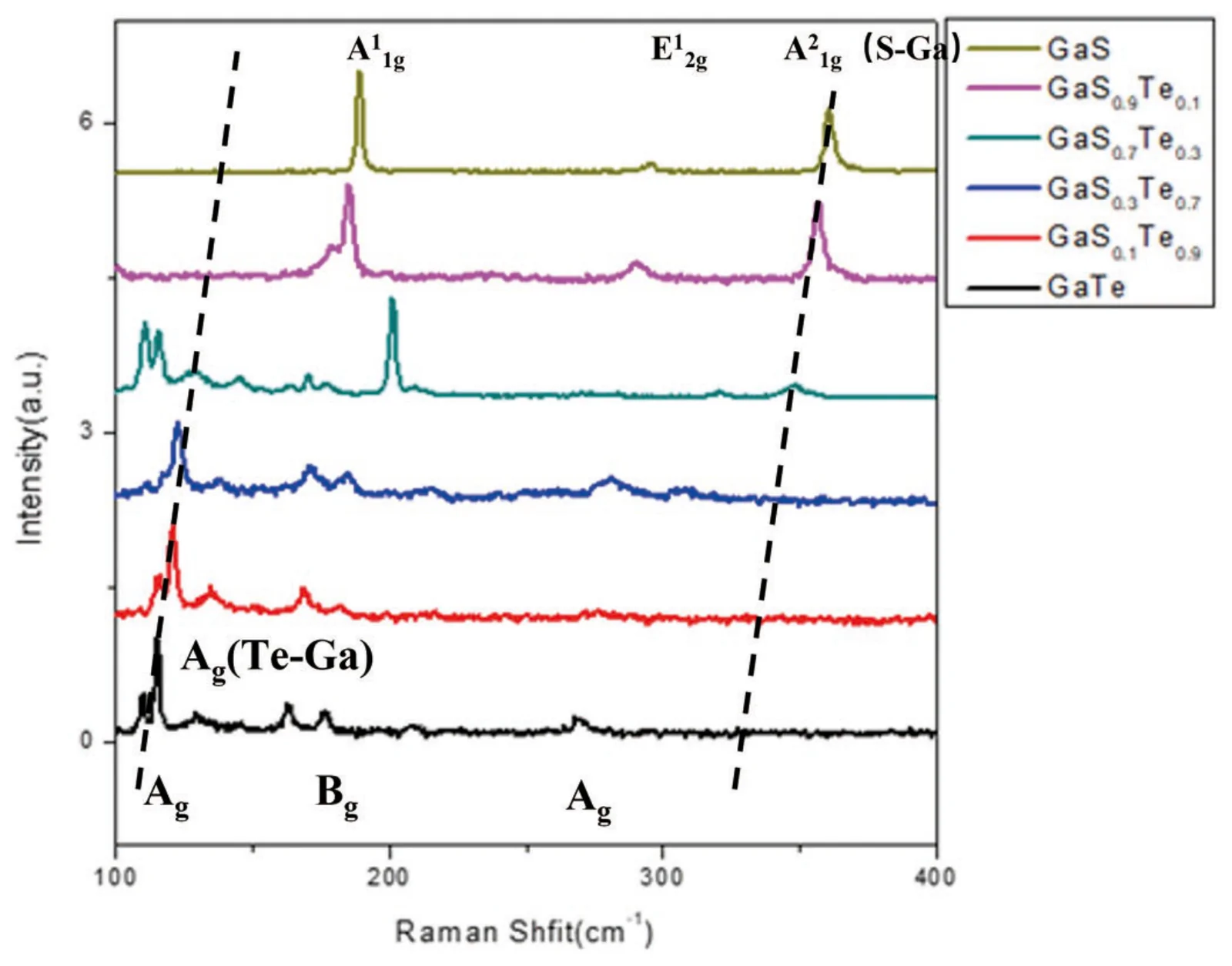
Raman spectrum of single GaS_*x*_Te_1−*x*_ (0.1⩽x⩽0.9). The molecular vibration mode changed from Te-Ga to S-Ga.

Raman spectroscopy was performed using a 532 nm laser as the excitation source to characterize the Raman features of GaS_*x*_Te_1−*x*_ alloy micro-/nanosheets with different compositions. As shown in Figure 4, the Raman spectrum of GaS exhibits three characteristic vibrational modes: A^1^_1*g*_, E^1^_2*g*_, and A^2^_1*g*_, consistent with previously reported Raman spectra of layered GaS [12,15,16,17,19,20,21,22]. The Raman spectrum of GaTe displays two characteristic modes: A_*g*_ and B_*g*_, at the bottom [15,16,17,19,20].

With increasing x, the intensity of the characteristic A_g_ mode of GaTe at approximately 129 cm^−1^ gradually decreases, while the GaS-related vibrational modes (around 360 cm^−1^) become increasingly prominent. These changes indicate that as the S content increases, the lattice vibrational characteristics progressively evolve from GaTe-like behavior to GaS-like behavior.

Raman scattering originates from photon–phonon interactions and reflects the vibrational properties of the crystal lattice. In GaS_*x*_Te_1−*x*_ alloy micro-/nanosheets, the Te–Ga-related Ag mode and the S–Ga-related A^2^_1*g*_ mode are located in the low-frequency and high-frequency regions, respectively. As the S/Te ratio varies, the corresponding vibrational modes undergo shifts in position and changes in intensity, reflecting the composition-dependent lattice vibrations in this alloy system.

### 3.3. Photoluminescence Properties of Te-Rich GaS_x_Te_*1−*x_ Alloy Nanosheets

**Figure 5:**
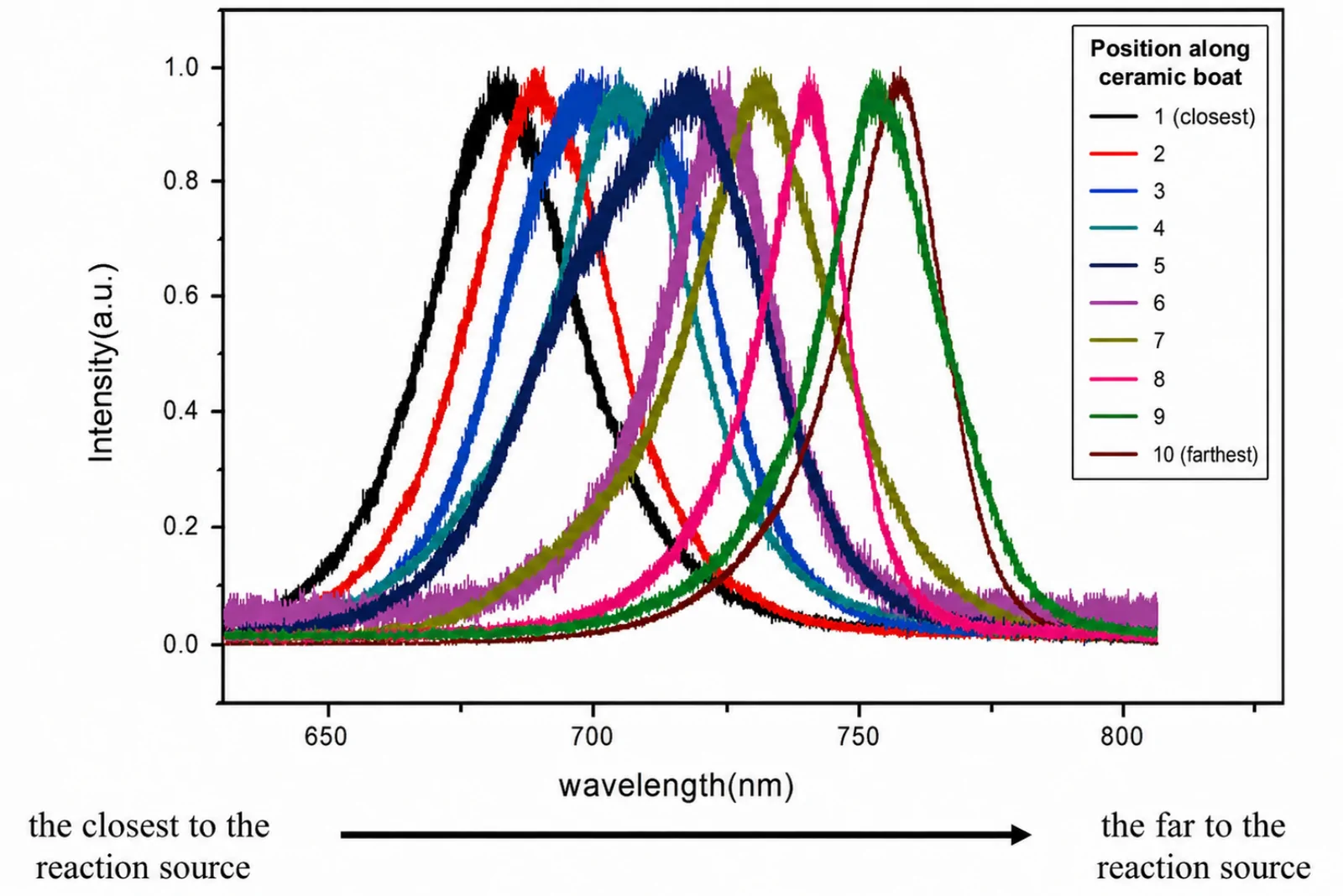
Under excitation of 532 nm laser, PL spectrum of GaS_*x*_Te_1−*x*_ Alloy micro-nanosheets for the molar ratio of m_*GaS*_:m_*GaTe*_ = 0.4:0.6.

Photoluminescence measurements were performed on GaS_*x*_Te_1−*x*_ alloy micro-/ nanosheets with compositions in the range of 0.3⩽x⩽0.5 using a 532 nm laser as the excitation source. Samples with different nominal compositions all exhibited position-dependent emission in the red-to-near-infrared region. For the precursor molar ratio m_*GaS*_:m_*GaTe*_ = 0.3:0.7, the PL peak varied over a range of approximately 660–760 nm; for the ratio 0.5:0.5, the PL peaks were mainly distributed in the range of 695–755 nm. Overall, samples with 0.3⩽x⩽0.5 exhibited broad red-to-near-infrared emission ranges, with PL peak positions shifting continuously along the substrate position. Taking GaS_0.4_Te_0.6_ as an example, Figure 5 shows PL spectra measured at different positions on the same growth substrate. As the measurement position moved from the end near the reaction source toward the end farther from the source, the PL peaks shifted continuously from approximately 680 to 760 nm, indicating distinct spatially dependent luminescence characteristics along the substrate direction.

### 3.4. Photoluminescence Properties of S-Rich GaS_x_Te_*1−*x_ Alloy Nanosheets

Photoluminescence measurements were performed on GaS_*x*_Te_1−*x*_ alloy micro-/ nanosheets with compositions in the range of 0.6⩽x⩽0.9 using a 355 nm laser as the excitation source. Compared with the Te-rich samples, the luminescence characteristics of samples in this composition range changed significantly with increasing S content. For 0.6⩽x⩽0.7, both green and red-to-near-infrared emission was observed at different positions on the same substrate. When the S proportion was further increased to 0.8⩽x⩽0.9, the PL peaks gradually shifted toward the green wavelength region around 520–530 nm.

**Figure 6:**
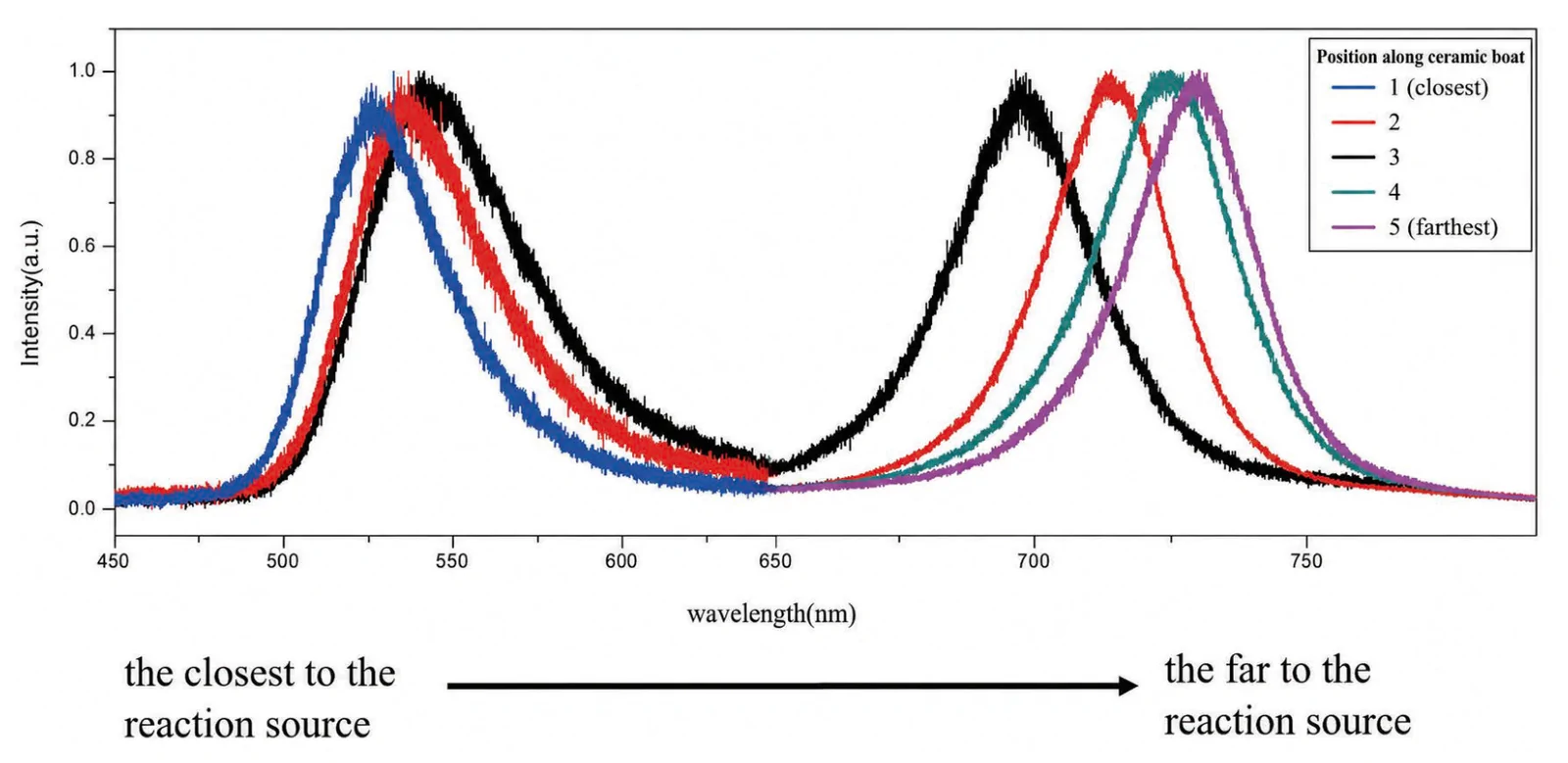
Under excitation of 355 nm laser, PL spectrum of GaS_*x*_Te_1−*x*_ Alloy micro-nanosheets for the molar ratio of m_*GaS*_:m_*GaTe*_ = 0.6:0.4.

Figure 6 shows PL spectra of the GaS_0.6_Te_0.4_ sample measured at different positions along the growth substrate. Samples near the reaction source end emitted mainly in the range of 525–540 nm, exhibiting green emission characteristics close to those of GaS; samples at the end farther from the source emitted mainly in the range of 695–730 nm, exhibiting red-to-near-infrared emission characteristics close to those of GaTe. Similar spatial variation trends were also observed in the GaS_0.7_Te_0.3_ sample. It should be noted that, for the GaS_0.6_Te_0.4_ sample, the PL peaks were mainly distributed in two relatively separated emission regions (green and red), rather than showing a single emission peak that shifted continuously across the range of 525–730 nm. This result indicates that under conditions of intermediate S content, the local compositions at different positions on the same substrate may straddle two luminescence regimes—one dominated by GaS characteristics and the other by GaTe characteristics.

**Figure 7:**
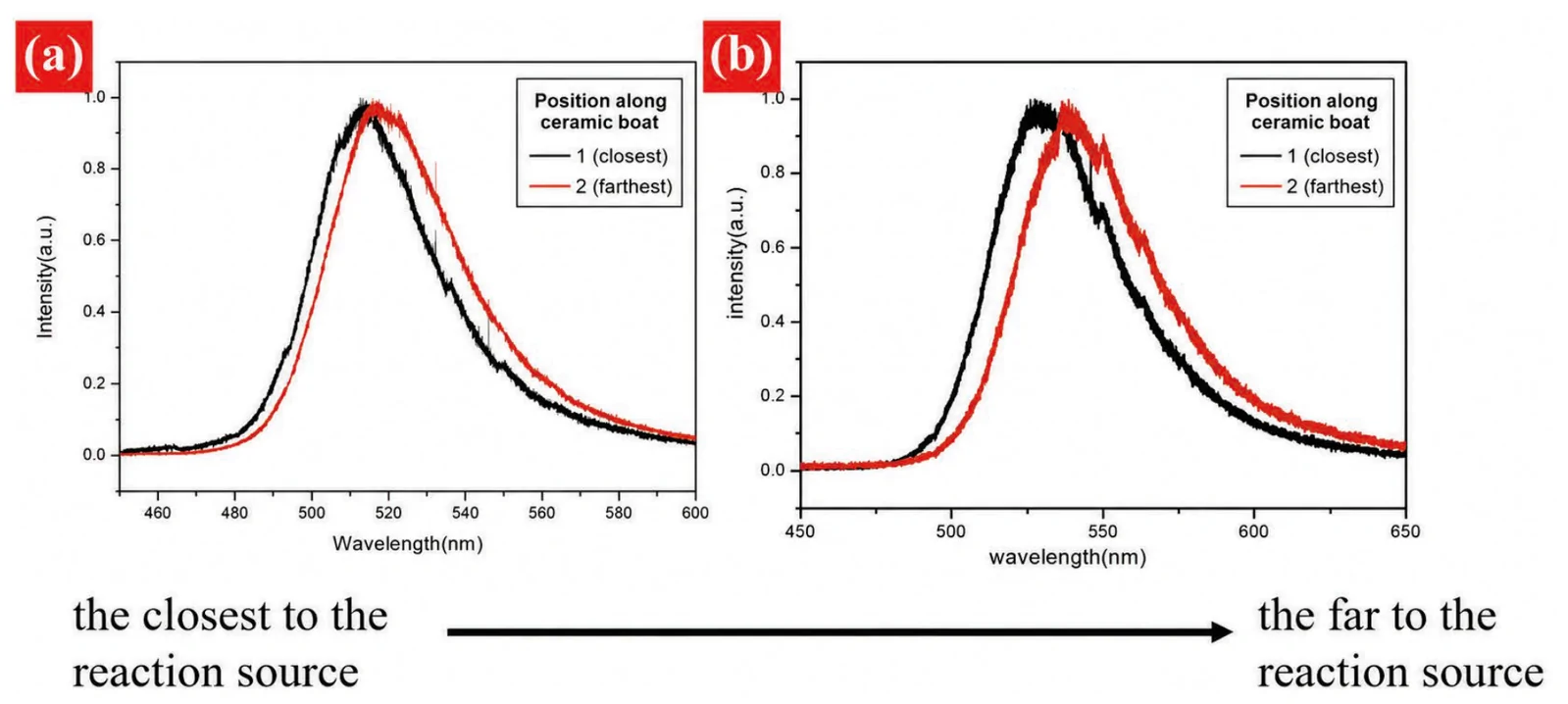
Under excitation of 355 nm laser, (**a**) PL spectrum of GaS_*x*_Te_1−*x*_ Alloy micro-nanosheets for the molar ratio of m_*GaS*_:m_*GaTe*_ = 0.8:0.2, (**b**) PL spectrum of GaS_*x*_Te_1−*x*_ Alloy micro-nanosheets for the molar ratio of m_*GaS*_:m_*GaTe*_ = 0.9:0.1.

As the S proportion in the precursors further increased, the range of PL peak positions decreased significantly. As shown in Figure 7, the PL peaks of the GaS_0.8_Te_0.2_ sample were mainly located at approximately 530 nm, while those of the GaS_0.9_Te_0.1_ sample were located at approximately 520 nm, with relatively minor peak position variations along the substrate direction. This may be attributed to the fact that at high *S* contents, most regions of the substrate form alloy compositions close to the GaS end-member; the small amount of Te and its spatial variation are insufficient to induce a wide range of bandgap tuning, and thus the luminescence is predominantly governed by the GaS-related green emission channel.

To further quantitatively characterize the fluorescence efficiency of the selected GaS_0.9_Te_0.1_ powder, its absolute photoluminescence quantum yield (PLQY) was measured using an FLS1000 fluorescence spectrophotometer. Under 355 nm excitation, the absolute PLQY was determined to be 9.49%. Together with its relatively stable emission centered around 520 nm, the measured PLQY provides further characterization of the photophysical properties of GaS_0.9_Te_0.1_ as a fluorescent powder for latent fingermark visualization.

## 4. GaS_*x*_Te_1−*x*_ (m_*GaS*_:m_*GaTe*_ = 0.9:0.1) Nanosheet Powder for Qualitative and Quantitative Analysis of Latent Fingermark Development

### 4.1. Qualitative Analysis

Compared with intermediate-composition samples exhibiting broad or dual-band emission characteristics, the PL peaks of GaS_*x*_Te_1−*x*_ (m_*GaS*_:m_*GaTe*_ = 0.9:0.1) are mainly concentrated around 520 nm, with relatively minor variations in peak position along the substrate. This indicates that the luminescence is predominantly governed by the GaS-related green emission channel, with a relatively concentrated emission band. The material exhibits distinct green fluorescence around 520 nm, which facilitates clear differentiation between latent fingermark ridges and the substrate background. Considering its emission stability, color distinguishability, and imaging consistency, GaS_0.9_Te_0.1_ nanosheets were selected as the latent fingermark development powder for subsequent experiments, and their practical performance was further evaluated through development experiments on various substrates.

**Figure 8:**
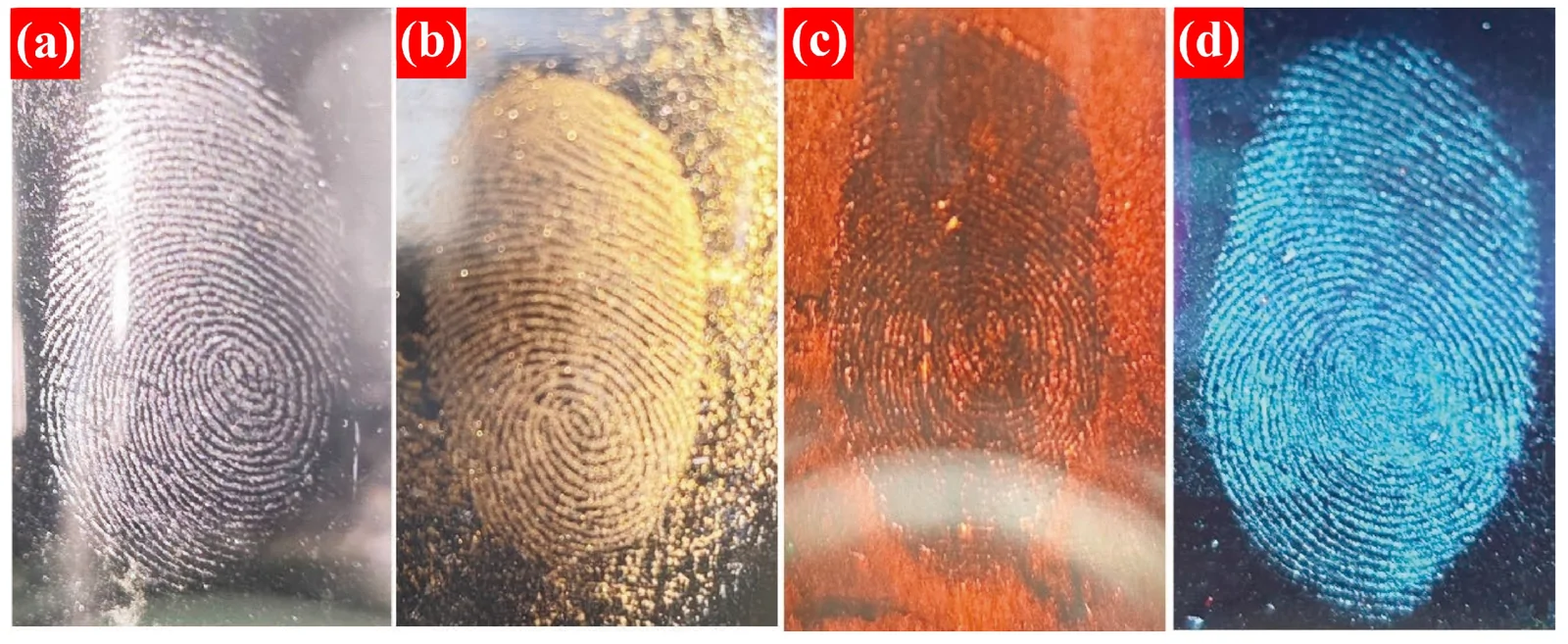
Development of latent fingermarks on glass using (**a**) silver powder, (**b**) gold powder, (**c**) red fluorescent powder, and (**d**) GaS_*x*_Te_1−*x*_ nanosheet powder.

As shown in Figure 8, silver powder produced relatively complete overall ridge patterns with continuous papillary ridges; gold powder revealed the main ridge patterns but exhibited local unevenness in powder deposition; and the red fluorescent powder showed strong background signals, resulting in poor differentiation between the ridges and the background. In contrast, the green fluorescence generated by GaS_*x*_Te_1−*x*_ (m_*GaS*_:m_*GaTe*_ = 0.9:0.1) nanopowder provided strong contrast with the transparent glass background, making the central ridge patterns and local details relatively easy to distinguish. The overall performance was comparable to that of silver powder, while providing more prominent fluorescence visibility. Therefore, this material demonstrates favorable fingermark development performance on glass surfaces.

**Figure 9:**
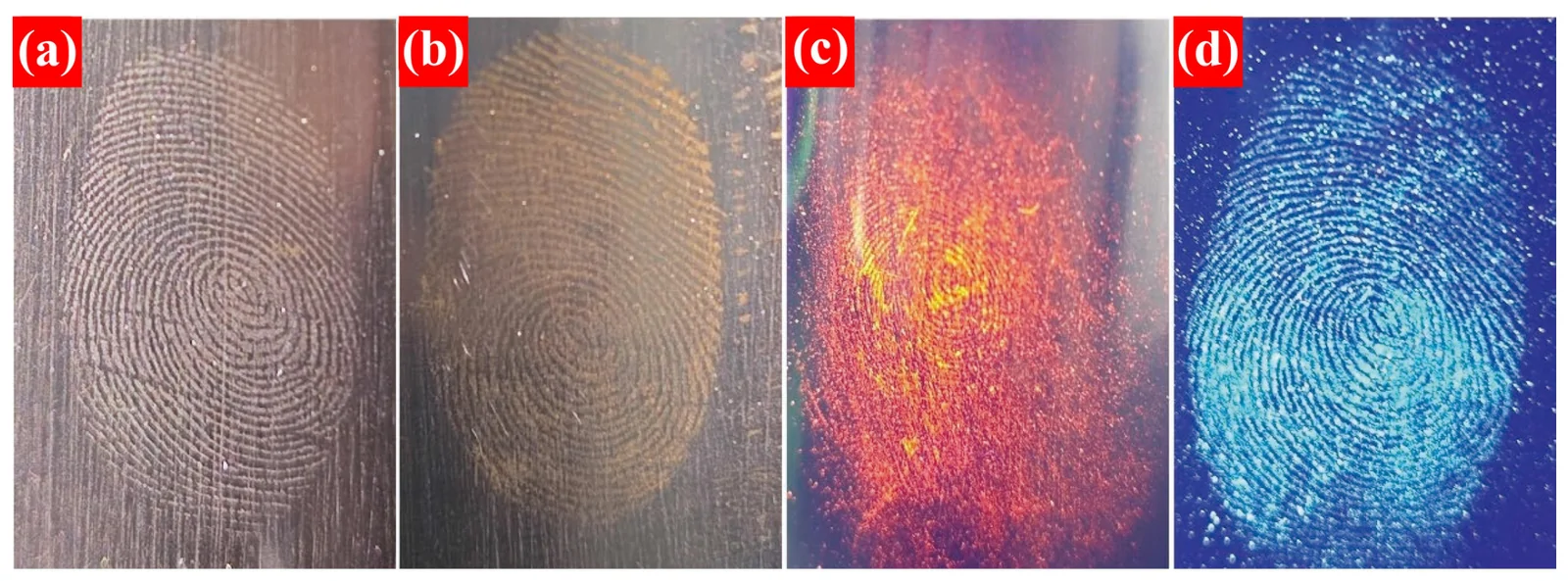
Development of latent fingermarks on stainless steel using (**a**) silver powder, (**b**) gold powder, (**c**) red fluorescent powder, and (**d**) GaS_*x*_Te_1−*x*_ nanosheet powder.

As shown in Figure 9, the reflective nature of stainless steel reduced the contrast of images developed with silver powder. Although gold powder revealed fingermark outlines, local ridge details were still affected by background reflection. The red fluorescent powder exhibited excessive powder deposition, which compromised detail discernibility. The green fluorescence of GaS_*x*_Te_1−*x*_ (m_*GaS*_:m_*GaTe*_ = 0.9:0.1) nanopowder enhanced the visual difference between the fingermark region and the metallic background, with overall ridge patterns and some endpoint and bifurcation features being relatively clear. However, some powder deposition was still observed in furrows and background areas, indicating that selective adhesion requires further improvement. Overall, the advantage of this material on stainless steel surfaces is mainly reflected in its fluorescence contrast.

**Figure 10:**
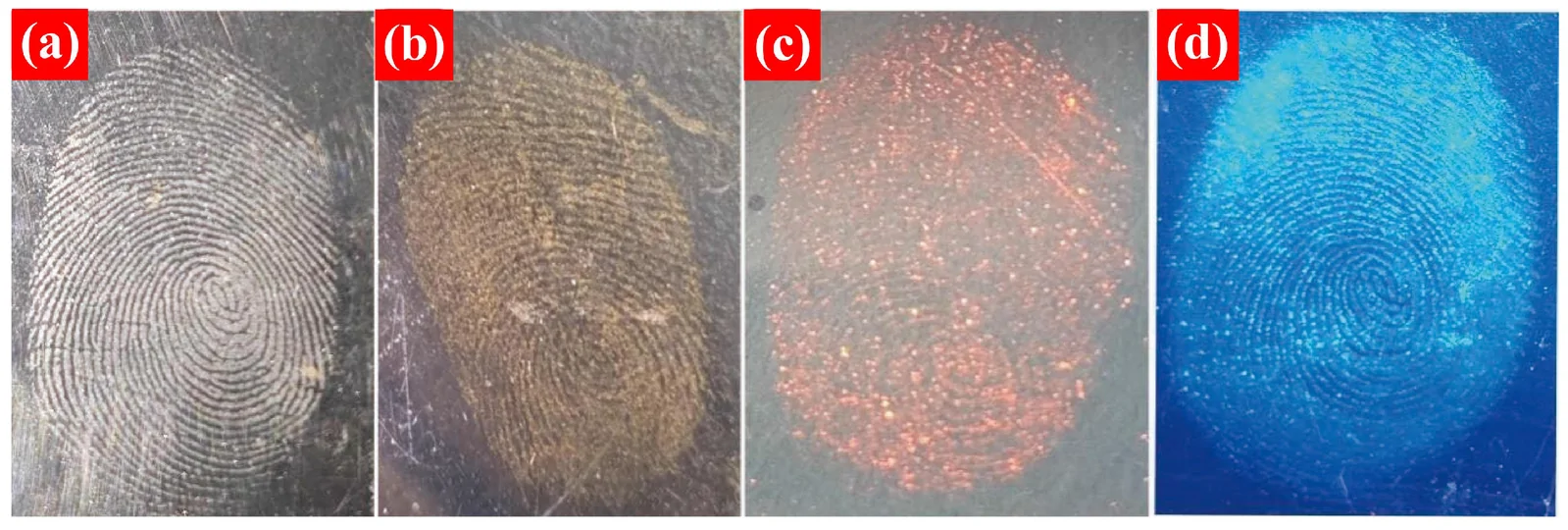
Development of latent fingermarks on plastic using (**a**) silver powder, (**b**) gold powder, (**c**) red fluorescent powder, and (**d**) GaS_*x*_Te_1−*x*_ nanosheet powder.

As shown in Figure 10, both silver powder and gold powder revealed the overall ridge patterns on plastic surfaces relatively clearly, with silver powder showing better ridge continuity. The red fluorescent powder exhibited noticeable background speckling and lower detail resolution. GaS_*x*_Te_1−*x*_ (m_*GaS*_:m_*GaTe*_ = 0.9:0.1) nanopowder produced clearly outlined green fluorescent fingermarks, but local powder aggregation and furrow deposition rendered some ridge boundaries less distinct. Compared with metallic powders, its advantage lies in fluorescence visibility, while it has not yet demonstrated a clear advantage in ridge fidelity; further optimization of powder dosage and dispersibility is required.

**Figure 11:**
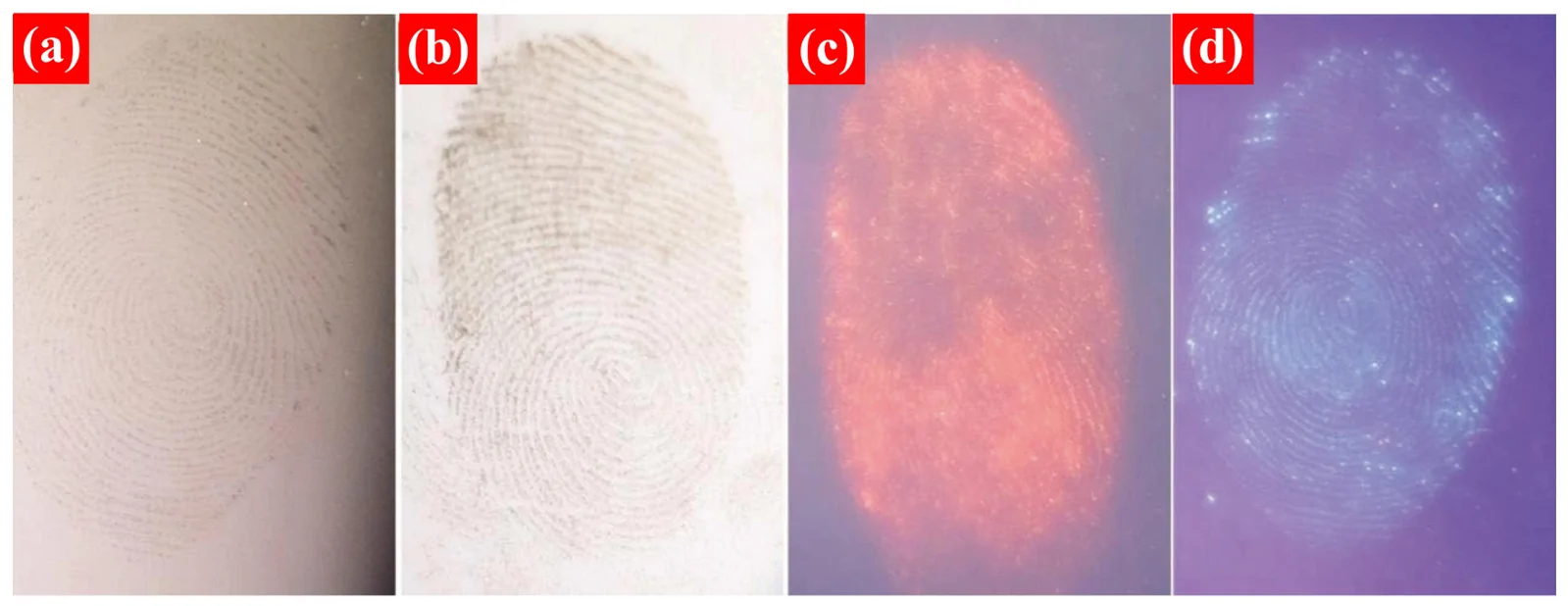
Development of latent fingermarks on plastic using (**a**) silver powder, (**b**) gold powder, (**c**) red fluorescent powder, and (**d**) GaS_*x*_Te_1−*x*_ nanosheet powder.

As shown in Figure 11, the color of silver powder was close to that of the light-colored ceramic background, resulting in low fingermark contrast. Although gold powder and red fluorescent powder revealed basic outlines, they suffered from local ridge loss or uneven background fluorescence. The green fluorescence generated by GaS_*x*_Te_1−*x*_ (m_*GaS*_:m_*GaTe*_ = 0.9:0.1) nanopowder provided strong contrast with the ceramic background, with relatively complete overall ridge patterns and superior ridge continuity and fidelity compared with the other three powders. Recent studies have shown that fluorescence-assisted latent fingermark development can be enhanced through different material-dependent mechanisms. For example, the enhanced performance of some fingermark development methods on rough non-porous substrates is primarily attributed to selective adhesion to fingermark residues rather than fluorescence intensity alone [37]. Aggregation-induced emission materials have also been used for latent fingerprint visualization by combining aggregation-state fluorescence with molecular-structure-dependent affinity toward organic fingermark residues [39].

Additionally, Oliveira et al. reported that fluorescent MCM-41@Ch@DnsGly nanoparticles can provide high-quality fingermark development on substrates with varying chemical compositions and surface properties; 94% of the developed images contained sufficient minutiae for comparison, with level 1 to level 3 features being discernible [44]. In contrast, the GaS_0.9_Te_0.1_ system investigated in this study represents a different material design strategy, namely, screening a fluorescent developer from composition-tunable two-dimensional semiconductor alloys based on its relatively stable emission around 520 nm, followed by direct application as a powder on several common non-porous substrates. The sheet-like morphology may provide a larger contact interface with fingermark residues, thereby promoting physical adhesion [45]. However, this interfacial mechanism was not directly quantified in the present study.

### 4.2. Quantitative Analysis

**Figure 12:**
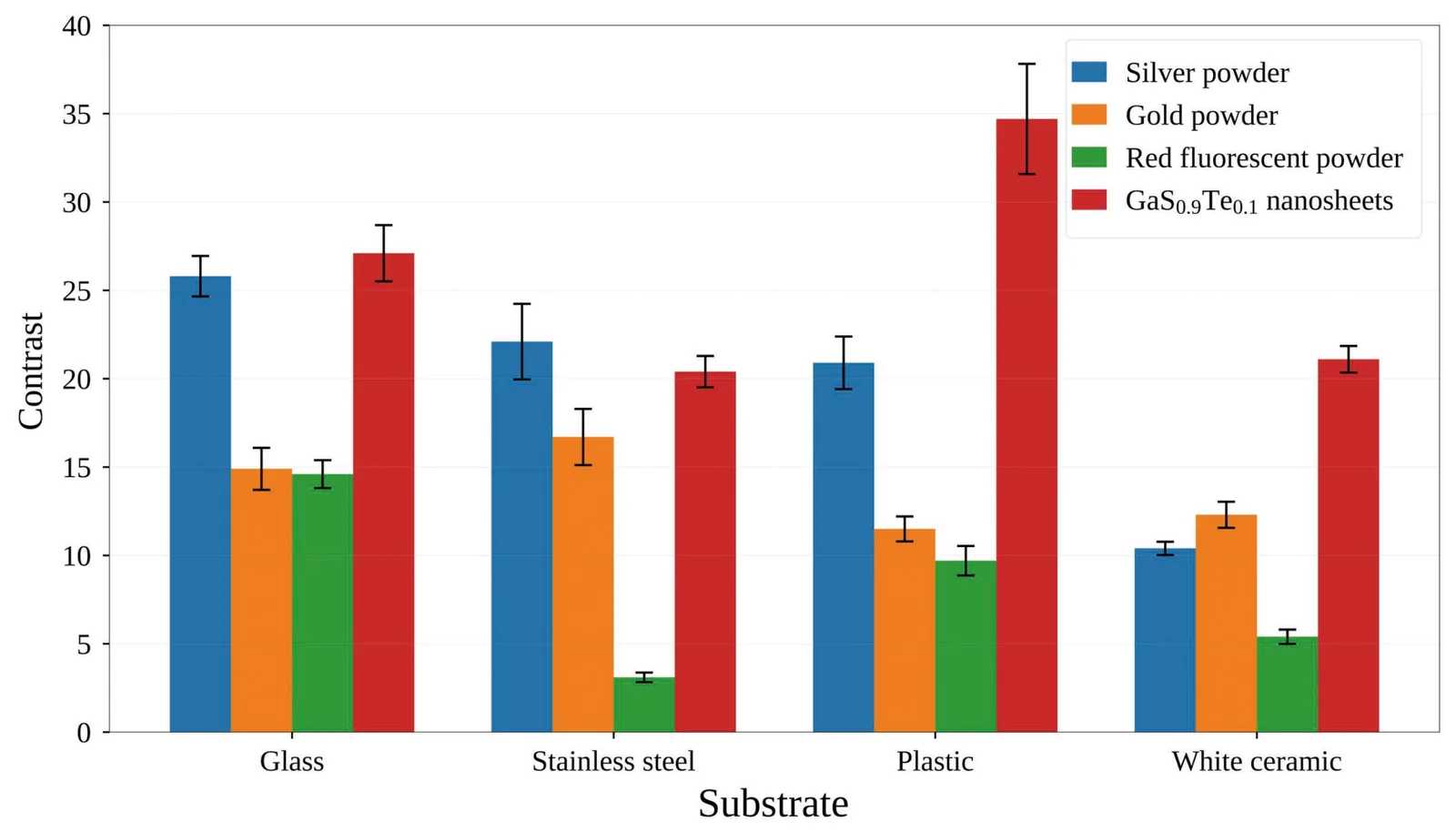
Bar chart of the contrast values of latent fingermarks developed with silver powder, gold powder, red fluorescent powder, and GaS_0.9_Te_0.1_ nanosheets on glass, stainless steel, plastic, and a white ceramic cup.

Quantitative contrast analysis was performed using five independently deposited latent fingermarks for each powder–substrate combination (n = 5), with results expressed as the mean ± standard deviation (SD), as shown in Figure 12. As shown in Table 2, one-way ANOVA revealed significant differences among the four development powders on all four substrates (glass: F(3,16) = 156.24, *p* < 0.001; stainless steel: F(3,16) = 186.44, *p* < 0.001; plastic: F(3,16) = 199.21, *p* < 0.001; white ceramic: F(3,16) = 608.44, *p* < 0.001).

On glass substrates, the mean contrast of GaS_0.9_Te_0.1_ nanosheets was 27.10 ± 1.59, slightly higher than that of silver powder (25.80 ± 1.15). However, Tukey’s HSD post hoc test indicated that this difference was not statistically significant (*p* = 0.358). In contrast, the contrast of GaS_0.9_Te_0.1_ was significantly higher than that of gold powder and red fluorescent powder (*p* < 0.001).

On stainless steel substrates, the mean contrast of GaS_0.9_Te_0.1_ nanosheets was 20.40 ± 0.89, while that of silver powder was 22.10 ± 2.14. Although the mean value for silver powder was slightly higher, the difference between the two powders was not statistically significant (*p* = 0.265). However, the contrast of GaS_0.9_Te_0.1_ was significantly higher than that of gold powder (*p* = 0.0038) and red fluorescent powder (*p* < 0.001).

The most pronounced advantage of GaS_0.9_Te_0.1_ was observed on plastic and white ceramic substrates. On plastic, the contrast of GaS_0.9_Te_0.1_ was 34.70 ± 3.12, significantly higher than that of silver powder (20.90 ± 1.49), gold powder (11.50 ± 0.71), and red fluorescent powder (9.70 ± 0.84) (all *p* < 0.001). Similarly, on white ceramic, the contrast of GaS_0.9_Te_0.1_ was 21.10 ± 0.75, significantly higher than that of silver powder (10.40 ± 0.37), gold powder (12.30 ± 0.74), and red fluorescent powder (5.40 ± 0.40) (all *p* < 0.001).

Overall, these quantitative results indicate that GaS_0.9_Te_0.1_ nanosheets provide contrast comparable to that of silver powder on glass and stainless steel, while demonstrating statistically significant advantages over the three reference powders on plastic and white ceramic. These findings are consistent with qualitative observations, suggesting that the primary advantage of GaS_0.9_Te_0.1_ is particularly evident on substrates where conventional powders offer relatively limited optical contrast.

Zhou et al. obtained a *D* value of 51.21 for sebum-rich fingermarks on frosted plastic substrates [37], whereas the highest D value obtained for GaS_0.9_Te_0.1_ on plastic in the present study was 34.7. Comparison of *D* values on different substrates can reflect the contrast of developed latent fingermarks, suggesting that further improvement in particle–residue selectivity may contribute to enhancing the contrast of GaS_0.9_Te_0.1_ nanosheets. Various quantitative metrics have been proposed for fluorescence-based fingermark evaluation. Wang et al. developed a spectroscopic method combining fluorescence intensity, chromaticity, and luminance to more comprehensively characterize fluorescence contrast [42]. In contrast, the present study employed local grayscale differences between ridges and furrows, which provide a direct and reproducible comparison among the four tested powders under identical experimental conditions but do not fully describe fluorescence color or spectral contrast. Therefore, the *D* values in this study are most appropriately interpreted as an internal comparative measure rather than a universal performance metric. Future integration of grayscale analysis with fluorescence spectral evaluation and standardized forensic image quality assessment may enable more rigorous comparisons with other fluorescent fingermark developers.

The quantitative results showed that GaS_0.9_Te_0.1_ nanosheets provided high fingermark-development contrast on different substrates. As shown in Figure 11, the contrast values on glass, plastic, and a white ceramic cup were 27.1, 34.7, and 21.1, respectively, all of which were the highest among the four development powders. The advantage was most pronounced on plastic, where the contrast was substantially higher than that obtained with silver powder, gold powder, and red fluorescent powder. On the stainless-steel cup, the contrast of GaS_0.9_Te_0.1_ nanosheets was 20.4, slightly lower than the 22.1 obtained with silver powder but still higher than the values obtained with gold powder and red fluorescent powder. This indicates that the nanosheets also produced a clear ridge-to-background contrast on metallic substrates.

Overall, the mean contrast of GaS_0.9_Te_0.1_ nanosheets across the four substrate types was 25.83, exceeding the corresponding mean values of 19.80, 13.85, and 8.20 for silver powder, gold powder, and red fluorescent powder, respectively, thereby demonstrating good applicability across different substrates. The relative error ranged from 3.56% to 8.99%, with a mean relative error of 5.69%, which was generally lower than those of the other three powders and indicated good measurement repeatability. In summary, GaS_0.9_Te_0.1_ nanosheets not only produced high development contrast on most substrates but also showed clear advantages on plastic and white ceramic, where conventional powders were comparatively less effective. These findings demonstrate their potential as a fluorescent powder for latent fingermark development.

### 4.3. Development of Aged Latent Fingermarks and Temporal Stability of Developed Fingermarks

**Figure 13:**
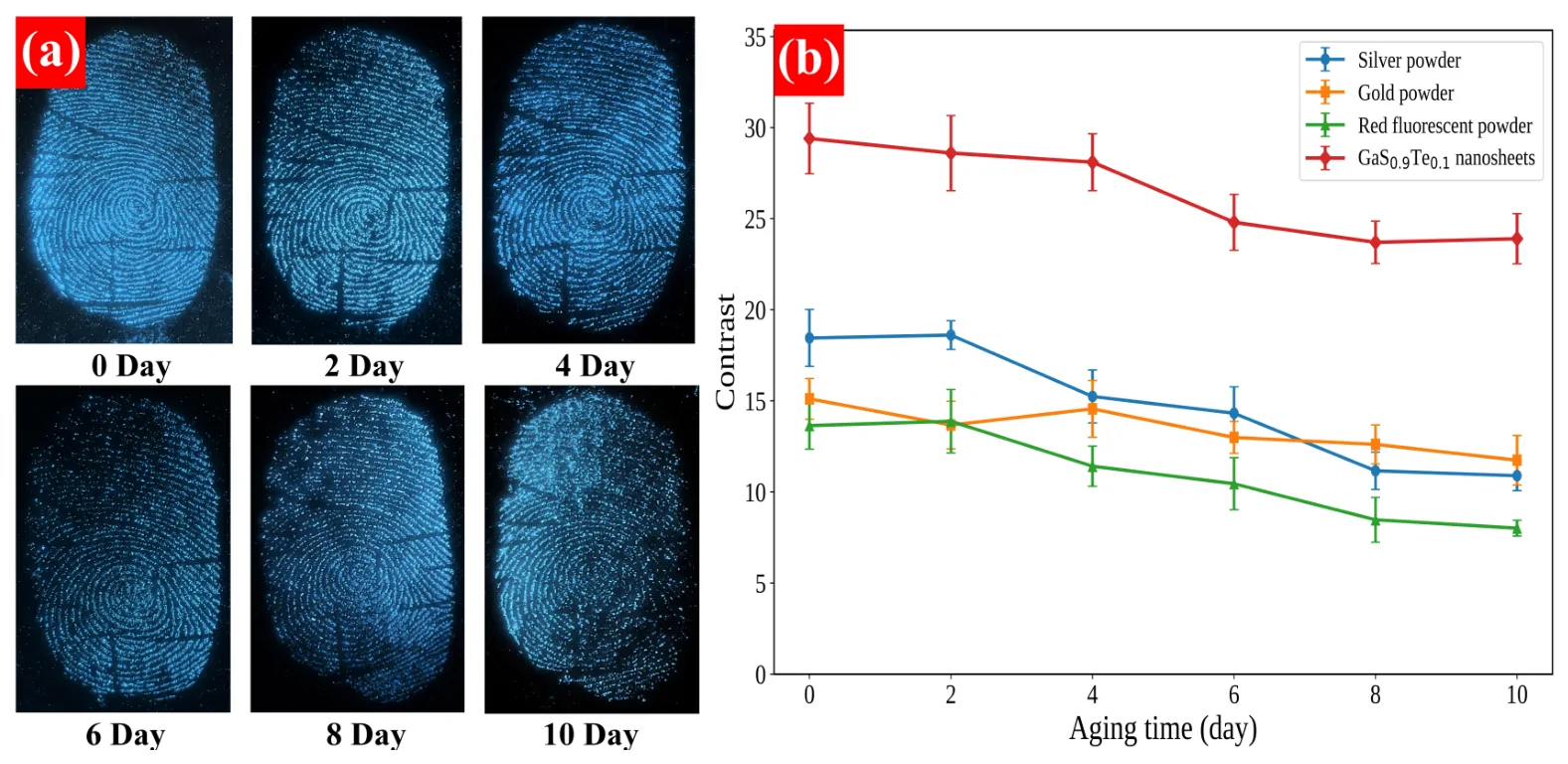
(**a**) Development results of latent fingermarks of different aging times (0, 2, 4, 6, 8, 10 days) using GaS_0.9_Te_0.1_ nanosheets; (**b**) Contrast variation curves of GaS_0.9_Te_0.1_ nanosheets, silver powder, gold powder, and commercial red fluorescent powder for latent fingermarks of different aging times.

As shown in Figure 13, the practical performance of GaS_0.9_Te_0.1_ nanosheets was further evaluated by developing latent fingermarks aged for 0, 2, 4, 6, 8, and 10 days comparing them with silver powder, gold powder, and commercial red fluorescent powder. With increasing aging time, the contrast obtained with all four powders showed a declining trend. However, throughout the entire aging period, GaS_0.9_Te_0.1_ consistently maintained the highest contrast, decreasing only from 29.4 on day 0 to 23.9 on day 10. In contrast, the corresponding values for silver powder, gold powder, and red fluorescent powder on day 10 were 10.89, 11.74, and 8.01, respectively. GaS_0.9_Te_0.1_ nanosheets retained approximately 81.3% of their initial contrast after 10 days, with relative errors ranging from 4.91% to 7.21%. These results indicate that GaS_0.9_Te_0.1_ nanosheets maintain relatively stable fingerprint development performance during aging and show potential for visualizing aged latent fingerprints.

**Figure 14:**
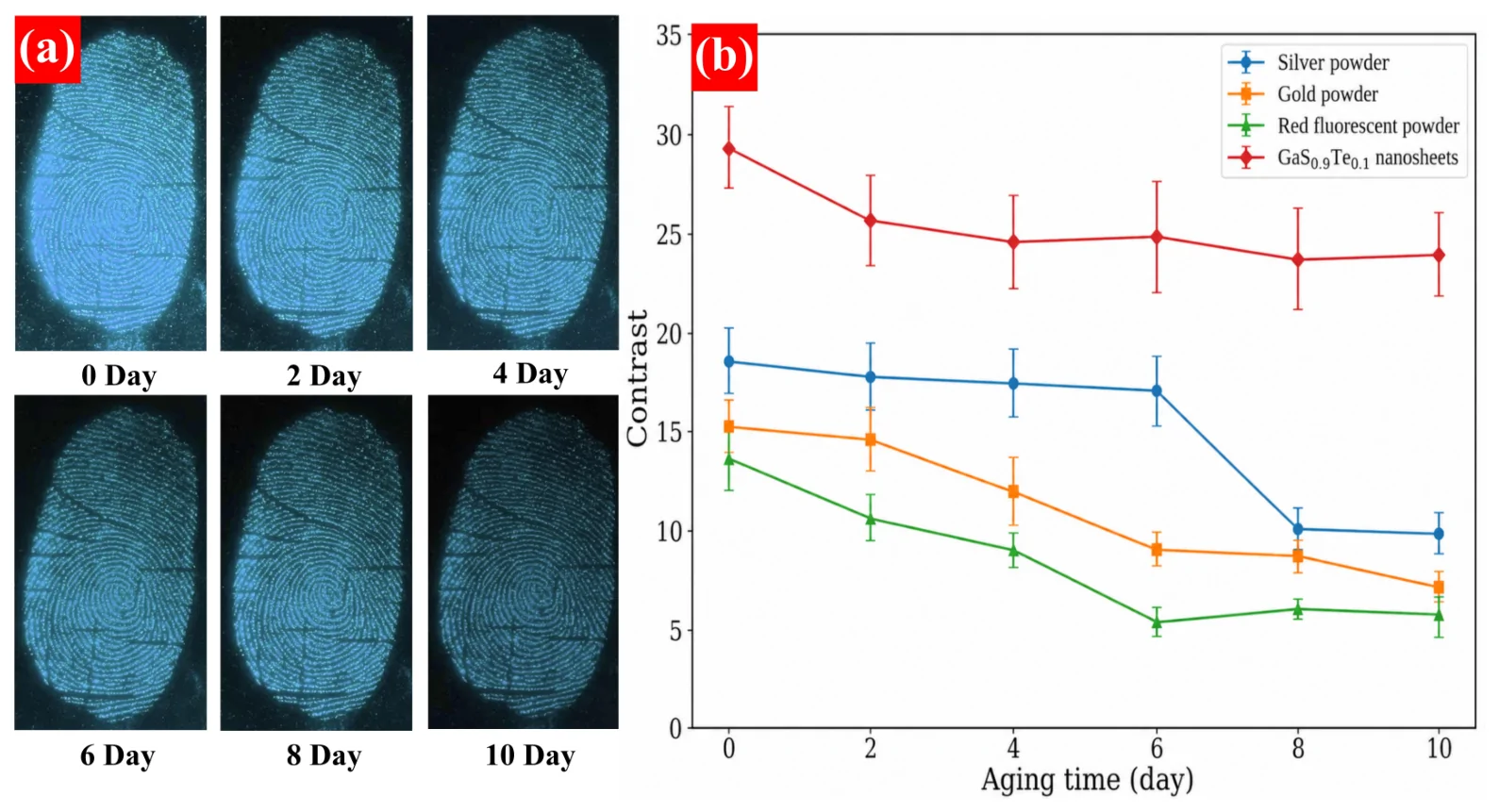
(**a**) Images of latent fingermarks at different time points (0, 2, 4, 6, 8, 10 days) after development with GaS_0.9_Te_0.1_ nanosheets; (**b**) Contrast variation curves of GaS_0.9_Te_0.1_ nanosheets, silver powder, gold powder, and commercial red fluorescent powder at different time points after fingermark development.

As shown in Figure 14, the stability of developed latent fingermarks was further evaluated by continuously observing fingermarks developed with different powders for 10 days. With increasing storage time, the contrast of all groups decreased, but GaS_0.9_Te_0.1_ nanosheets exhibited better stability, with contrast decreasing from 29.4 on day 0 to 23.9 on day 10, retaining approximately 81.3% of the initial value. In contrast, silver powder, gold powder, and red fluorescent powder retained only approximately 54.0%, 47.7%, and 43.8% of their initial contrast, respectively, on day 10. These results indicate that latent fingermarks developed with GaS_0.9_Te_0.1_ nanosheets possess favorable temporal stability.

## 5. Conclusions

This study characterized composition-dependent structural and optical properties of GaS_*x*_Te_1−*x*_ alloy nanosheets. SEM and EDS analyses confirmed the sheet-like morphology and relatively uniform elemental distribution of the synthesized GaS_*x*_Te_1−*x*_ materials, while Raman and photoluminescence measurements revealed a gradual evolution from GaTe-like to GaS-like characteristics with increasing *S* content. GaS_0.9_Te_0.1_ was identified as the most suitable composition for fingermark visualization. Owing to its relatively stable emission around 520 nm with limited variation in peak position and an absolute photoluminescence quantum yield of 9.49%, GaS_0.9_Te_0.1_ was selected as a fluorescent powder for latent fingermark development. Furthermore, GaS_0.9_Te_0.1_ provided effective fluorescent fingermark development on several non-porous substrates. Clear ridge patterns and favorable ridge–background contrast were obtained on glass, plastic, stainless steel, and light-colored ceramic, with particularly prominent performance on glass, plastic, and ceramic surfaces. Quantitative comparisons confirmed its potential as a complementary fluorescent developer. The average contrast value of GaS_0.9_Te_0.1_ across the four substrates was 25.83, higher than the corresponding values of silver powder (19.80), gold powder (13.85), and commercial red fluorescent powder (8.20). Furthermore, stability experiments on developed fingermarks and experiments involving the development of aged fingermarks indicated that the material maintained detectable development performance within a 10-day observation window. Future work should focus on improving particle dispersibility and selective adhesion to fingermark residues, evaluating batch-to-batch synthesis reproducibility and long-term storage stability, and extending the tests to multiple donors and a broader range of substrate types.

## Figures and Tables

**Table 1:** ICP-MS results and calculated elemental contents for the GaS_0.9_Te_0.1_ sample with the nominal composition.

Sample	Ga (wt%)	S (wt%)	Te (wt%)
GaS_0.9_Te_0.1_	66.3145	27.456	6.2295

**Table 2:** Quantitative comparison of local contrast for latent fingermarks developed with different powders on non-porous substrates.

Substrate	Silver Powders	Gold Powders	Red Fluorescent Powders	${\mathrm{GaS}}_{x}{\mathrm{Te}}_{1-x}$ ($m_{\mathrm{GaS}}$:$m_{\mathrm{GaTe}}$ = 0.9:0.1) Nanosheets	F(3,16)	*p* Value
Glass	25.80 ± 1.15 ^a^	14.90 ± 1.19 ^b^	14.60 ± 0.79 ^b^	27.10 ± 1.59 ^a^	156.24	<0.001
Stainless steel	22.10 ± 2.14 ^a^	16.70 ± 1.59 ^b^	3.10 ± 0.27 ^c^	20.40 ± 0.89 ^a^	186.44	<0.001
Plastic	20.90 ± 1.49 ^b^	11.50 ± 0.71 ^c^	9.70 ± 0.84 ^c^	34.70 ± 3.12 ^a^	199.21	<0.001
White ceramic	10.40 ± 0.37 ^c^	12.30 ± 0.74 ^b^	5.40 ± 0.40 ^d^	21.10 ± 0.75 ^a^	608.44	<0.001

Data are presented as mean ± SD (n = 5 independently deposited latent sweat fingermarks per condition). Within each row, values marked with different superscript letters are significantly different (one-way ANOVA followed by Tukey’s HSD post-hoc test, *p* < 0.05).

## Data Availability

The data presented in this article and the codes used are available upon request to the corresponding authors.
